# Headspace analysis of new psychoactive substances using a Selective Reagent Ionisation-Time of Flight-Mass Spectrometer

**DOI:** 10.1016/j.ijms.2013.12.009

**Published:** 2014-03-01

**Authors:** W. Joe Acton, Matteo Lanza, Bishu Agarwal, Simone Jürschik, Philipp Sulzer, Kostiantyn Breiev, Alfons Jordan, Eugen Hartungen, Gernot Hanel, Lukas Märk, Chris A. Mayhew, Tilmann D. Märk

**Affiliations:** a IONICON Analytik GmbH, Eduard-Bodem-Gasse 3, 6020 Innsbruck, Austria; b Lancaster Environment Centre, Lancaster University, LA1 4YQ Lancaster, UK; c Institut für Ionenphysik und Angewandte Physik, Leopold-Franzens Universität Innsbruck, Technikerstr. 25, 6020 Innsbruck, Austria; d School of Physics and Astronomy, University of Birmingham, Edgbaston, Birmingham B15 2TT, UK

**Keywords:** PTR-MS, SRI-TOF-MS, New psychoactive substances, Drug detection, Branching ratios

## Abstract

The rapid expansion in the number and use of new psychoactive substances presents a significant analytical challenge because highly sensitive instrumentation capable of detecting a broad range of chemical compounds in real-time with a low rate of false positives is required. A Selective Reagent Ionisation-Time of Flight-Mass Spectrometry (SRI-ToF-MS) instrument is capable of meeting all of these requirements. With its high mass resolution (up to *m*/Δ*m* of 8000), the application of variations in reduced electric field strength (*E*/*N*) and use of different reagent ions, the ambiguity of a nominal (monoisotopic) *m*/*z* is reduced and hence the identification of chemicals in a complex chemical environment with a high level of confidence is enabled. In this study we report the use of a SRI-ToF-MS instrument to investigate the reactions of H_3_O^+^, O_2_^+^, NO^+^ and Kr^+^ with 10 readily available (at the time of purchase) new psychoactive substances, namely 4-fluoroamphetamine, methiopropamine, ethcathinone, 4-methylethcathinone, N-ethylbuphedrone, ethylphenidate, 5-MeO-DALT, dimethocaine, 5-(2-aminopropyl)benzofuran and nitracaine. In particular, the dependence of product ion branching ratios on the reduced electric field strength for all reagent ions was investigated and is reported here. The results reported represent a significant amount of new data which will be of use for the development of drug detection techniques suitable for real world scenarios.

## Highlights

• Headspace SRI-ToF-MS analysis of 10 new psychoactive substances.
• Product ions following ionisation with H_3_O^+^, NO^+^, O_2_^+^ and Kr^+^ are shown.
• The effect of reduced electric field strength on product ion branching ratios is presented.
• The compiled data should act as a resource for future method development.

## Graphical abstract

**Figure:**
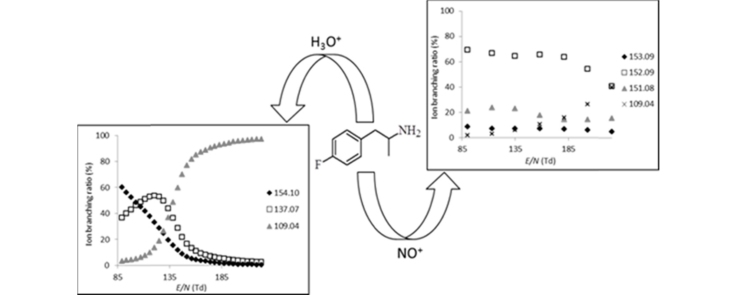

## 1 Introduction

The abuse of drugs is an important issue affecting today's society. Although many drug species are controlled by law, a market for new psychoactive substances (i.e. legal highs, research chemicals, and designer drugs) which are not controlled by drug legislation, has recently emerged. These readily available drugs are increasingly being used as substitutes for prohibited drugs, especially by those who are looking for a high, but who do not wish to commit a criminal act [1].

A review of the current literature shows that most new psychoactive substances have received little scientific interest, especially substances new to the market. For many of these compounds the only up-to-date source of information (e.g. synthesis, purity, side-effects, etc.) is to be found online in user forums [2,3] and no data on properties like proton affinity, ionisation energy, etc. are available. As new psychoactive substances regularly enter the market, it is important that broad-based analytical methods exist which have the ability to rapidly detect them, without the need for major changes in operational procedures. This rapid identification is especially important if a user has taken an unidentified drug and requires urgent medical treatment. Gas chromatography–mass spectrometry (GC–MS) has traditionally been used for the identification of drugs [4], providing both high selectivity and sensitivity. This comes at the expenses of fast analysis, making a real-time and therefore on-the-spot analysis impossible. Chemical test strips and ion mobility spectrometry (IMS) are much faster methods of analysis, but these have a limited selectivity [5].

Proton-Transfer-Reaction-Time of Flight-Mass-Spectrometry (PTR-ToF-MS), which relies on the use of H_3_O^+^ as the reagent ion, provides both a rapid detection capability and a high sensitivity (pptv within seconds). In addition, the soft ionisation capabilities of PTR-ToF-MS generally avoid significant fragmentation of the analytes which enables drug identification with a high level of confidence (low rate of false positives). However, relying on a nominal *m/z* makes unambiguous identification impossible. High mass resolution instruments provide higher confidence in assignment, but still isomeric compounds cannot be ruled out. In addition to changing operational parameters, e.g. the voltage applied to the drift tube, the recently developed Selective Reagent Ionisation (SRI) technology, [6,7] to change the reagent ion and hence alter the ion-molecule chemistry in the drift tube of a PTR-ToF-MS, has significantly increased the instrument's selectivity, making it a multidimensional technique [8]. Given that we have used SRI in this study, we will not refer to the instrument as PTR-ToF-MS, but as a Selective Reagent Ionisation-Time of Flight-Mass Spectrometry (SRI-ToF-MS) instrument to reflect this multidimensional use.

Previous studies have illustrated the applicability of SRI-ToF-MS to the detection of several illicit and controlled prescription drugs [9] and numerous other threat substances, such as explosives [10–12], chemical warfare agent simulants and toxic industrial compounds [13–15]. In addition, it has been shown that for some explosives, selectivity can be enhanced by changing the voltage applied to the drift tube (e.g. with the use of H_3_O^+^ as the reagent ion, by increasing reduced electric field strengths *E*/*N* – the ratio of the electric field, *E*, to the buffer gas number density, *N*, in the drift tube – the protonated parent molecule signals of TNT and TNB are increased) [12].

**Fig. 1:**
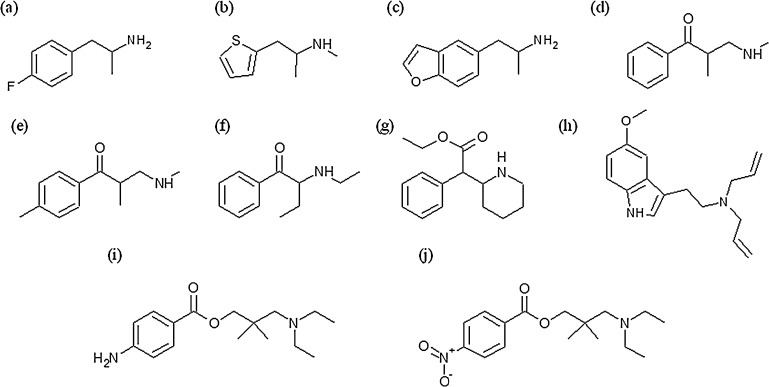
Chemical structures of (a) 4-fluoroamphetamine, (b) methiopropamine, (c) 5-(2-aminopropyl)benzofuran, (d) ethcathinone, (e) 4-methylethcathinone, (f) N-ethylbuphedrone, (g) ethylphenidate, (h) 5-MeO-DALT, (i) dimethocaine and (j) nitracaine.

In this paper a detailed study of the principle product ions observed following reactions of H_3_O^+^, NO^+^, O_2_^+^ and Kr^+^ with a number of new psychoactive substances, namely 4-fluoroamphetamine, methiopropamine, ethcathinone, 4-methylethcathinone, N-ethylbuphedrone, ethylphenidate, 5-MeO-DALT, dimethocaine, 5-(2-aminopropyl)benzofuran and nitracaine (for structural information see Fig. 1) is reported. In particular, the effects of *E*/*N* on the fragmentation pathways are also discussed in detail. These datasets, which provide information on the exact *m*/*z* and *E*/*N* dependence for all abundant fragments and with all four reagent ions, respectively, should help in the development of a highly selective analytical technique for drug detection based on SRI-ToF-MS suitable for real world scenarios.

## 2 Experimental

All new psychoactive substance samples were analysed using a PTR-TOF 8000 (IONICON Analytik GmbH, Austria) equipped with a SRI capability, thus allowing a change in the reagent ion used for chemical ionisation from H_3_O^+^ to O_2_^+^, NO^+^ or Kr^+^. The reagent ions and the resulting product ions are separated and detected using a ToF mass analyser. PTR-ToF-MS and SRI have both been described in detail in previous publications [6,7,16,17] and therefore they will only be briefly discussed here.

For the production of H_3_O^+^ ions water vapour from a reservoir of pure water enters a hollow cathode discharge source. Following ionisation and a series of ion-molecule reactions the resulting H_3_O^+^ ions are directed into the drift tube by an applied voltage gradient. Within the drift tube proton transfer reactions will take place only with those chemical species (M) that have a proton affinity (PA) greater than that of water (PA(H_2_O) = 691 kJ mol^−1^) [18]. This could either be via non-dissociative proton transfer:

(1) H_3_O + M → MH^+^ + H_2_O

and/or via dissociative proton transfer:

(2) H_3_O^+^ + M → [M − *A*]H^+^ + *A* + H_2_O

where *A* represents an elimination of a molecule from the transient protonated parent molecule.

For the production of O_2_^+^, NO^+^ and Kr^+^, water vapour is replaced by oxygen, an oxygen/nitrogen mix and krypton, respectively. If exothermic, the reactions with NO^+^, O_2_^+^ and Kr^+^ may proceed via charge transfer, which may also be either non-dissociative:

(3) X^+^ + M → M^+^ +X

where X^+^ represents the reagent ion, and/or dissociative:

(4) X^+^ + M → [M − *B*]^+^ +*B* + X

resulting in the elimination of *B* from the parent ion. NO^+^ has the lowest recombination energy (RE) of these three reagent ions (9.3 eV) and therefore may only charge transfer to neutral species whose ionisation energies (IE) are less than 9.3 eV. However, reaction of NO^+^ and a molecule may also take place via a chemical reaction, for example hydride abstraction:

(5) NO^+^ + M → [M − H]^+^ + HNO

Reaction with NO^+^ may result in an adduct ion being formed:

(6) NO^+^ + M + *C* → MNO^+^ + *C*

where *C* represents a third body (the buffer gas) that is required to remove some of the energy resulting from the association, without which the adduct would dissociate rapidly.

The *E*/*N* value used can be rapidly adjusted by changing the voltage applied across the drift tube; this enables the investigation of fragmentation pathways potentially offering a more selective method for compound identification. In the reported experiments the *E*/*N* value was varied between 85 and 225 Td (1 Td = 10^−21^ V m^2^) when H_3_O^+^, NO^+^ and O_2_^+^ were used as reagent ions and between 45 and 115 Td for the investigations using Kr^+^. The 10 new psychoactive substances studied, legal in most European countries at the time of purchase, were obtained from various online vendors. These new psychoactive substances studied were 4-fluoroamphetamine, methiopropamine, ethcathinone, 4-methylethcathinone, N-ethylbuphedrone, ethylphenidate, 5-MeO-DALT, dimethocaine, 5-(2-aminopropyl)benzofuran and nitracaine. Most can be classified as cathinones or piperazines, which include a large list of analogues of amphetamines, with psychoactive and stimulant effects [19,20]. Most of the chemicals were supplied in powder (or crystal) form. However, two were supplied in tablet form (4-fluoroamphetamine and ethcathinone). Therefore, before any measurements were taken these two compounds were first crushed into a fine powder. This ensured that all samples were of comparable surface area. All of the supplied chemicals were used with no purification. In addition to the detection of the advertised new psychoactive substance analysis with SRI-ToF-MS enables the detection of most solvents, synthetic reagents and intermediates, but not “extenders” (usually low cost inorganics such as sodium bicarbonate), however due to low costs of production and tough competition, with rankings in online forums, the use of these “extenders” is less likely in new psychoactive substances than in illegal drugs.

The following procedure was adopted for the dynamic headspace sampling of all 10 chemicals with H_3_O^+^, NO^+^ and O_2_^+^ as the reagent ions. A few mg of the drug were added to a glass vial, the vial was then sealed with a septum. An inlet and an outlet line (1/16th inch PEEK, internal diameter 1 mm, VICI AG International) were inserted through the septum to allow for dynamic headspace sampling. The inlet line was connected to a charcoal filter so that purified laboratory air entered the vials. The outlet line was directly connected to the heated sampling line of the SRI-ToF-MS instrument. Samples were heated to a temperature between 60 °C and 100 °C and the sampling line and drift tube were maintained at a temperature of 110 °C in order to minimise adsorption of the analytes onto the surface and memory effects.

Once the dynamic headspace concentration had equilibrated, an investigation of the reaction processes as a function of *E*/*N* began. For H_3_O^+^ the *E*/*N* value was increased from 85 to 225 Td in steps of 5 Td. A similar range in the reduced electric field was used for NO^+^ and O_2_^+^, but *E*/*N* was increased in ∼25 Td steps, as no significant changes in the product ion branching ratios were observed for smaller variations in *E*/*N*. For Kr^+^ the method was similar to the above with the only exception being that helium was used as buffer gas, instead of charcoal filtered air. This was required to prevent loss of Kr^+^ via charge transfer to the main components of air [7]. The use of helium as the buffer gas necessitated restricting the range of the Kr^+^ *E*/*N* study to a maximum value of 115 Td, to prevent plasma formation in the drift tube. Rapid switching between H_3_O^+^, NO^+^ and O_2_^+^ is possible (tens of seconds). However switching from H_3_O^+^ to Kr^+^ requires several (if not tens) of minutes, because of the need of a dry system [7].

Prior to the analysis of each chemical with a new reagent ion an empty vial was connected to the instrument. The *E*/*N* was varied over the same range used for the drugs, as described above, in order to provide background mass spectra which could be subtracted from those obtained when using the drug sample. For all measurements an integration time of 40 s was used.

## 3 Results and discussion

**Fig. 2:**
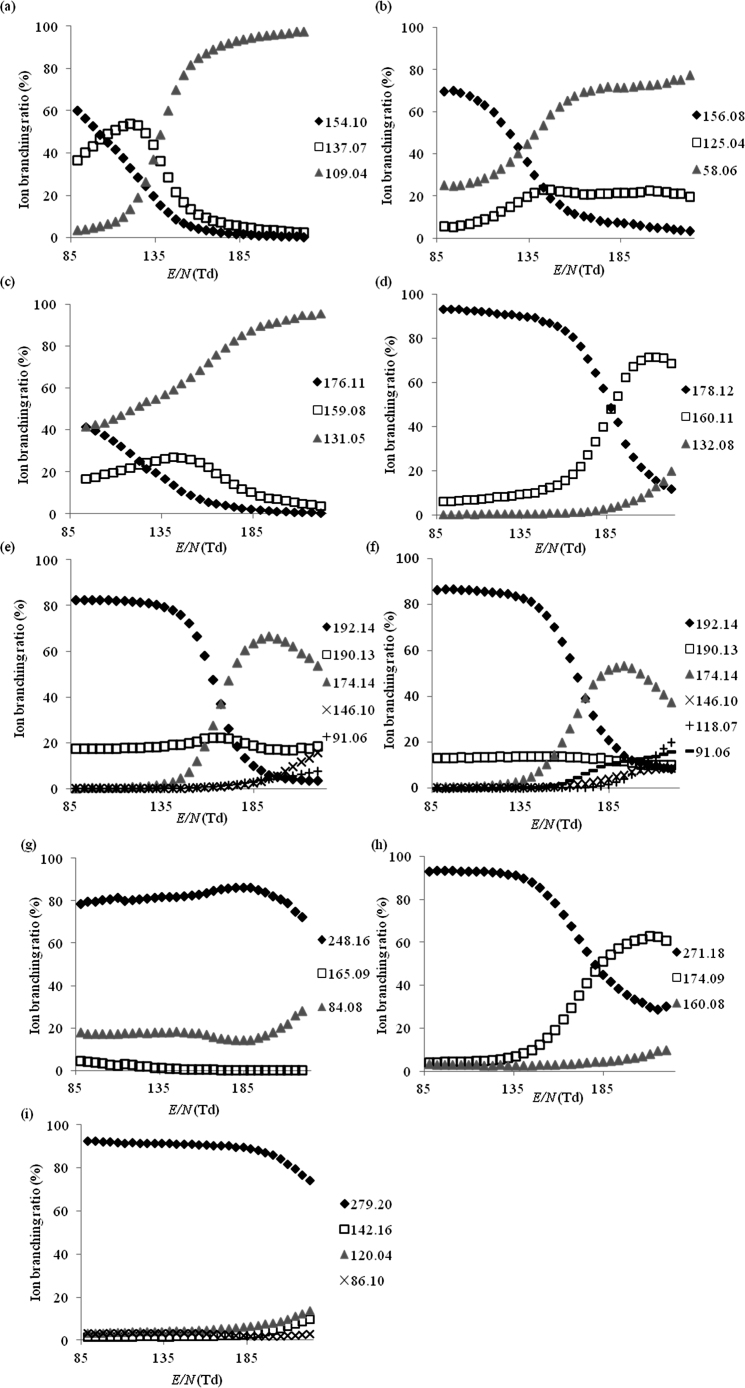
The variation of the percentage product ion branching ratios following the reactions of the various drug compounds with H_3_O^+^ as a function of *E*/*N* for (a) 4-fluoroamphetamine, (b) methiopropamine, (c) 5-(2-aminopropyl)benzofuran, (d) ethcathinone, (e) 4-methylethcathinone, (f) N-ethylbuphedrone, (g) ethylphenidate, (h) 5-MeO-DALT and (i) dimethocaine.

**Fig. 3:**
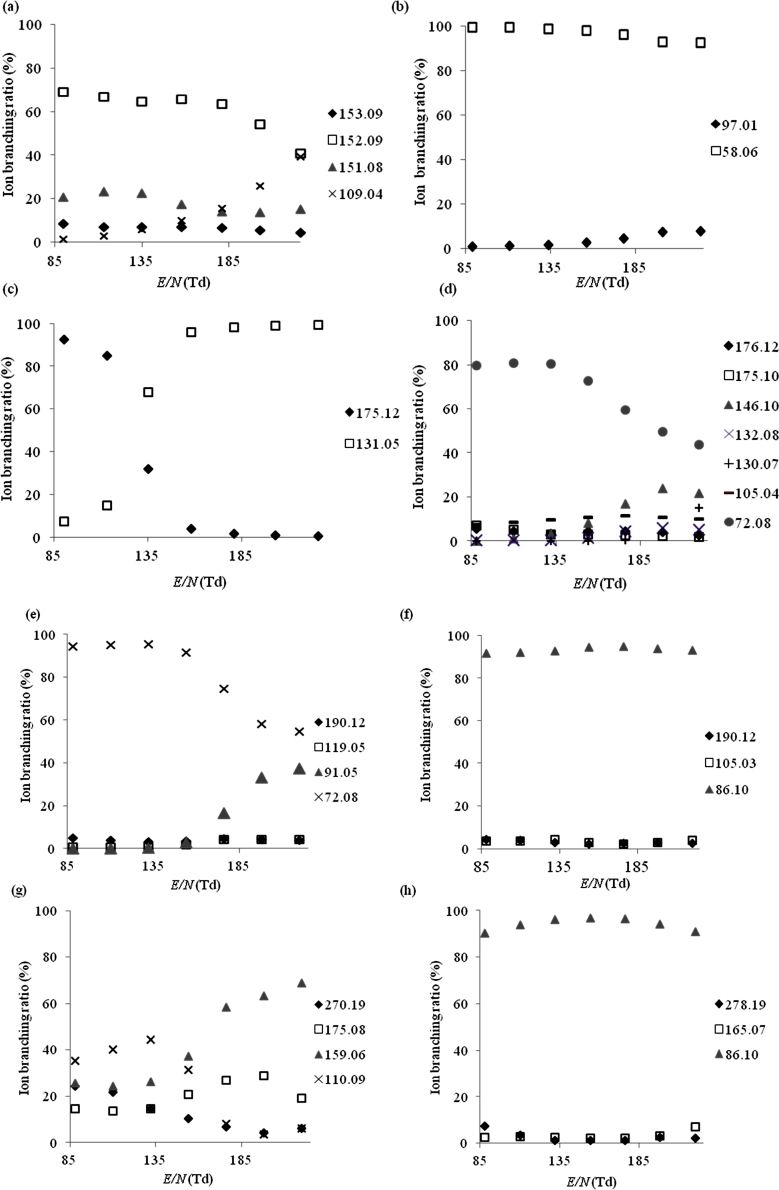
The variation of the percentage product ion branching ratios following the reactions of the drug species with NO^+^ as a function of *E*/*N* for (a) 4-fluoroamphetamine, (b) methiopropamine, (c) 5-(2-aminopropyl)benzofuran, (d) ethcathinone, (e) 4-methylethcathinone, (f) N-ethylbuphedrone, (g) 5-MeO-DALT and (h) dimethocaine.

**Fig. 4:**
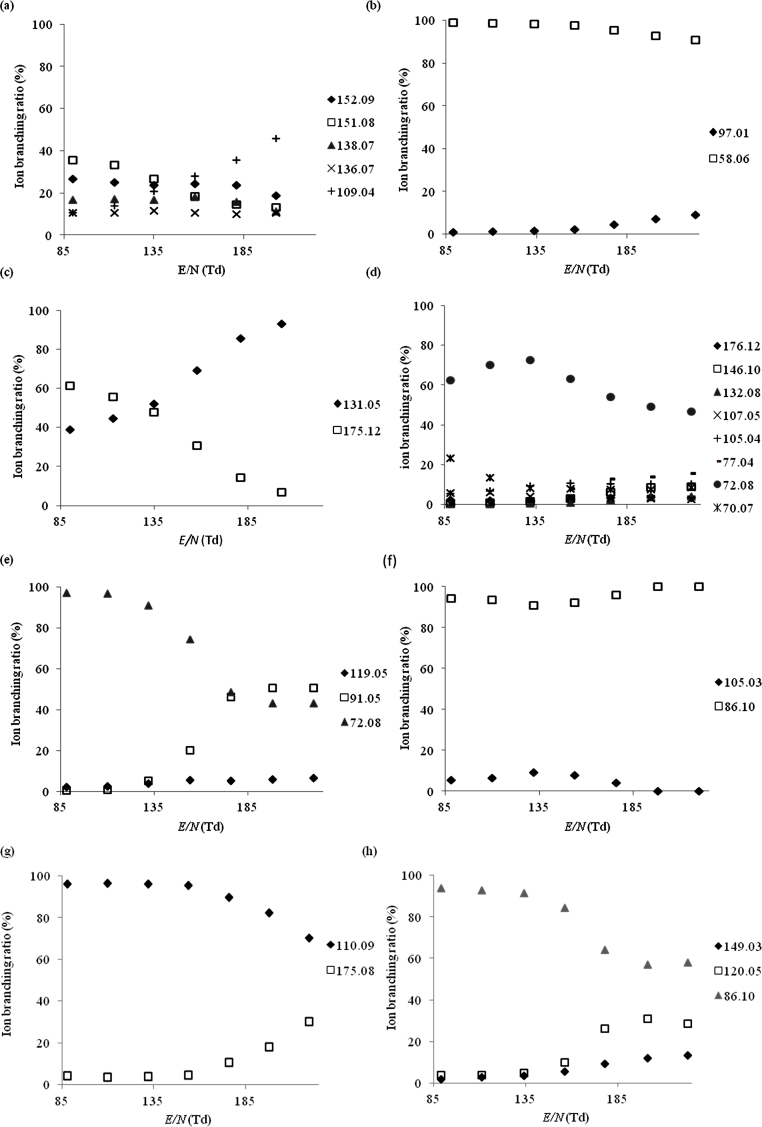
The variation of the percentage product ion branching ratios following the reactions of the drug species with O_2_^+^ as a function of *E*/*N* for (a) 4-fluoroamphetamine, (b) methiopropamine, (c) 5-(2-aminopropyl)benzofuran, (d) ethcathinone, (e) 4-methylethcathinone, (f) N-ethylbuphedrone, (g) 5-MeO-DALT and (h) dimethocaine.

**Fig. 5:**
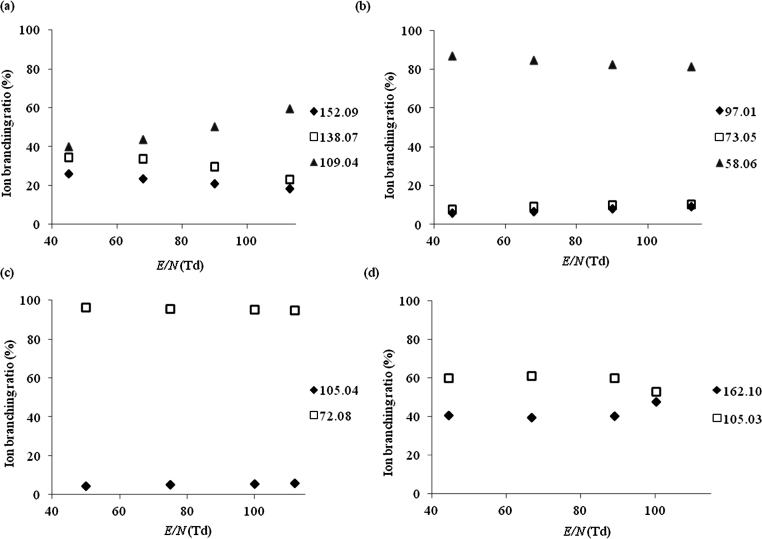
The variation of the percentage product ion branching ratios following the reactions of the drug species with Kr^+^ as a function of *E*/*N* for (a) 4-fluoroamphetamine, (b) methiopropamine, (c) ethcathinone and (d) N-ethylbuphedrone.

Product ion branching ratios for each drug species are summarised in Figs. 2–5 for reactions with H_3_O^+^, NO^+^, O_2_^+^ and Kr^+^, respectively. Only product ions which have branching ratios of greater than or equal to 3% within the *E*/*N* range studied have been included in the figures, because below this value there is greater uncertainty as to whether they belong to the drug or to an impurity.

Table 1 summarises the product ions and their branching ratios at a standard operating condition of 130 Td.

### 3.1 4-Fluoroamphetamine (4-FA)

#### 3.1.1 Reaction with H_3_O^+^

Fig. 2(a) shows the effect of changing *E*/*N* on product ion branching ratios when H_3_O^+^ is used as the reagent ion. It can be seen from the figure that the protonated parent molecule (*m*/*z* 154.10, [MH]^+^, C_9_H_13_FN^+^) dominates at *E*/*N* < 105 Td, whilst *m*/*z* 137.07 ([MH−NH_3_]^+^, C_9_H_10_F^+^) becomes the most abundant species between 105 and 135 Td. Above 135 Td the ion branching ratio of *m*/*z* 109.04 ([MH−C_2_NH_7_]^+^, C_7_H_6_F^+^) increases rapidly and becomes the dominant fragment ion. These ionic species have also been observed in a previous study using chemical ionisation–mass spectrometry [21]. However in that study the reagent gas used was methane, resulting in higher reaction energies than associated with the reagent ion H_3_O^+^ and hence increased fragmentation. Additional fragment ions were also detected in that earlier study, which we did not observe, the most notable at *m*/*z* 134 corresponding to [MH−HF]^+^.

#### 3.1.2 Reaction with NO^+^, O_2_^+^ and Kr^+^

When NO^+^ was used as the reagent ion, see Fig. 3(a), the dominant product ion observed across the whole *E*/*N* range was at *m*/*z* 152.09, assigned to [M−H]^+^ (C_9_H_11_FN^+^). The parent ion, *m*/*z* 153.09, was observed with an ion branching ratio which decreased from 9% to 4% as *E*/*N* was increased from 85 to 225 Td. An additional product ion was observed at *m*/*z* 109.04 corresponding to [M−C_2_H_6_N]^+^ (C_7_H_6_F^+^), which became the dominant product ion above 225 Td. A significant mass spectral peak assigned to an impurity was seen at *m*/*z* 124.03, which we assume resulted from non-dissociative charge transfer to 4-fluorobenzaldehyde (C_7_H_5_OF^+^), one of the reagents reported in the synthesis of the drug by the online community [22].

When O_2_^+^ was used as the reagent ion, Fig. 4(a), the parent ion peak was not observed. Instead we observed a product ion at *m*/*z* 152.09 which we attribute to the [M−H]^+^ ion, a fragmentation pathway which has been observed previously following the reaction of O_2_^+^ with chemical compounds [23], as well as product ions at *m*/*z* 138.07 ([M−CH_3_]^+^, C_8_H_9_FN^+^) and *m*/*z* 109.04 ([M−C_2_H_6_N]^+^, C_7_H_6_F^+^). As found with the NO^+^ study, we also observed a mass spectral peak at *m*/*z* 124.03 which we attribute to a reaction with an impurity.

Results similar to those found for O_2_^+^ and NO^+^ were observed for the reactions with Kr^+^, Fig. 5(a), i.e. the same principle product ions observed across the whole *E*/*N* range, namely ions at *m*/*z* 152.09 ([M−H]^+^, C_9_H_11_FN^+^), *m*/*z* 138.07 ([M−CH_3_]^+^, C_8_H_9_FN^+^) and *m*/*z* 109.04 ([M−C_2_H_6_N]^+^, C_7_H_6_F^+^). Again a mass spectral peak at *m*/*z* 124.03 (C_7_H_5_OF^+^) resulting from an impurity was observed.

### 3.2 Methiopropamine (MPA)

#### 3.2.1 Reaction with H_3_O^+^

The reaction of MPA with H_3_O^+^, Fig. 2(b), results in three main product ions, namely the protonated parent at *m*/*z* 156.08 ([MH]^+^, C_8_H_14_SN^+^), *m*/*z* 125.04 ([MH−CH_5_N]^+^, C_7_H_9_S^+^) and *m*/*z* 58.06 ([MH−C_5_H_6_S]^+^, C_3_H_8_N^+^). Significant spectral peaks were also observed at *m*/*z* 151.06 and *m*/*z* 63.03; the latter is attributed to NaBH_3_CN, which has been reported as a reagent in the synthesis of MPA [24], and this is supported by a distinctive isotopic pattern. The mass spectral peak at *m*/*z* 151.06 is unidentified.

At *E*/*N* values below 135 Td the protonated parent molecule was the dominant species, whilst both *m*/*z* 125.04 and *m*/*z* 58.06 increased with increasing *E*/*N* and *m*/*z* 58.06 (i.e. [MH−C_5_H_6_S]^+^, C_3_H_8_N^+^) became the most abundant product ion at values greater than 135 Td.

#### 3.2.2 Reactions with NO^+^, O_2_^+^ and Kr^+^

For both, NO^+^ and O_2_^+^, Figs. 3(b) and 4(b), respectively, *m*/*z* 58.06 ([M−C_5_H_5_S]^+^, C_3_H_8_N^+^) was the dominant product ion observed across the whole *E*/*N* range investigated. Above 155 Td the ion branching ratio of *m*/*z* 58.06 decreased slightly because a new ion channel opens resulting in a product ion at *m*/*z* 97.01, which we identify as [M−C_3_H_8_N]^+^ (C_5_H_5_S^+^). Two additional ions were observed for reactions with NO^+^ and O_2_^+^at *m*/*z* 111.99 and 169.02, tentitatively assigned to C_5_H_4_SO^+^ and C_7_H_7_SNO_2_^+^, respectively. The corresponding neutral species are known intermediates used in the synthesis of MPA [24]. Other, but unknown impurities resulted in ion signals observed at *m*/*z* 149.98 and *m*/*z* 137.09.

Product ions at *m*/*z* 58.06 and *m*/*z* 97.01 were also observed to result from reactions with Kr^+^ (Fig. 5(b)), with *m*/*z* 58.06 being again the dominant one. However, an additional fragment ion was observed at *m*/*z* 73.05 which is tentatively assigned to C_4_H_11_N^+^, an ion that could be formed either via cleavage at the thiophene ring (i.e. [M−C_4_H_2_S]^+^), or from charge transfer to *n*-BuNH_2_, a compound which has been reported as a reagent in the synthesis of MPA [24] but we consider this to be unlikely as it was not observed with reactions of NO^+^ and O_2_^+^.

### 3.3 5-(2-Aminopropyl)benzofuran (5-APB)

#### 3.3.1 Reaction with H_3_O^+^

In the case of reactions with H_3_O^+^ (Fig. 2(c)), the dominant product ion over the whole *E*/*N* range was at *m*/*z* 131.05 ([MH−C_2_H_7_N]^+^, C_9_H_7_O^+^). The protonated parent molecule ([MH]^+^, C_11_H_14_NO^+^) was observed at *m*/*z* 176.11. Another peak observed in the mass spectra was found at *m*/*z* 159.08. We assign this to [MH−NH_3_]^+^ (C_11_H_11_O^+^). The supplied sample of 5-APB showed major contributions from a series of impurities, mass spectral peaks observed which cannot be assigned to the drug, included *m*/*z* 119.03, *m*/*z* 101.02, and *m*/*z* 59.05 tentatively assigned to protonated benzofuran (C_8_H_7_O^+^), isopropenyl acetate (C_5_H_8_O_2_^+^), a solvent used in the synthesis of both 4-(2-aminopropyl)benzofuran (4-APB) and 6-(2-aminopropyl)benzofuran (6-APB), which are structural isomers of 5-APB [25], and acetone (C_3_H_7_O^+^), respectively.

#### 3.3.2 Reactions with NO^+^, O_2_^+^ and Kr^+^

Two product ions attributed to 5-APB, which resulted from the reaction with O_2_^+^ (Fig. 4(c)), i.e. *m*/*z* 175.12 ([M]^+^, C_11_H_13_NO^+^) and *m*/*z* 131.05 ([M−C_2_H_6_N]^+^, C_9_H_7_O^+^) were observed. In agreement with the study involving H_3_O^+^, the reaction of O_2_^+^ with the supplied sample of 5-APB also indicates that it is impure, i.e. mass spectral peaks observed at *m*/*z* 100.04 (ionised isopropenyl acetate, C_5_H_7_O_2_^+^) and at *m*/*z* 74.05 (an unknown compound). In the case of NO^+^ (Fig. 3(c)), the same two products ions were observed, but with different branching ratios. A significant impurity resulted in an ion being observed at *m*/*z* 147.96. As a result of considerable fragmentation no product ions could be confidently attributed to 5-APB following its reaction with Kr^+^.

### 3.4 Ethcathinone

#### 3.4.1 Reactions with H_3_O^+^

Following reaction with H_3_O^+^ (Fig. 2(d)), the dominant product ion observed at *E*/*N* values below 185 Td is the protonated parent molecule at *m*/*z* 178.12 ([MH]^+^, C_11_H_16_NO^+^). Both *m*/*z* 160.11 ([MH−H_2_O]^+^, C_11_H_14_N^+^) and *m*/*z* 132.08 ([MH−C_2_H_6_O]^+^, C_9_H_10_N^+^), were also observed and their associated branching ratios are found to increase with increasing *E*/*N*. Once *E*/*N* becomes greater than 185 Td, *m*/*z* 160.11 becomes the dominant product ion.

#### 3.4.2 Reactions with NO^+^, O_2_^+^ and Kr^+^

The dominant product ion observed from the dissociative charge transfer reaction with NO^+^ (Fig. 3(d)) was *m*/*z* 72.08, assigned to [M−C_7_H_5_O]^+^ (C_4_H_10_N^+^) which most probably is formed by α-cleavage at the ketone functional group. Other ions were observed at *m*/*z* 105.04 ([M−C_4_H_10_N]^+^, C_7_H_5_O^+^), *m*/*z* 146.10, ([M−CH_3_O]^+^, C_10_H_12_N^+^), *m*/*z* 175.10 ([M−H_2_]^+^, C_11_H_13_NO^+^) and *m*/*z* 176.12 ([M−H]^+^, C_11_H_14_NO^+^). At *E*/*N* > 175 Td the fragments *m*/*z* 132.08 ([M−C_2_H_5_O]^+^, C_9_H_10_N^+^) and *m*/*z* 130.07 ([M−C_2_H_7_O]^+^, C_9_H_8_N^+^) were also observed. The use of O_2_^+^ as the reagent ion (Fig. 4(d)) led to the formation of many of the same product ions as were identified with NO^+^, i.e. *m*/*z* 72.08 ([M−C_7_H_5_O]^+^, C_4_H_10_N^+^), *m*/*z* 105.04 ([M−C_4_H_10_N]^+^, C_7_H_5_O^+^), *m*/*z* 132.08 ([M−C_2_H_5_O]^+^, C_9_H_10_N^+^), *m*/*z* 146.10 ([M−CH_3_O]^+^, C_10_H_12_N^+^) and *m*/*z* 176.12 ([M−H]^+^, C_11_H_14_NO^+^), with the ion at *m*/*z* 72.08 again being the dominant fragment ion. Additional ions were seen at *m*/*z* 107.05 ([M−C_4_H_8_N]^+^, C_7_H_7_O^+^), *m*/*z* 77.04 ([M−C_5_H_10_NO]^+^, C_6_H_5_^+^) and *m*/*z* 70.07 ([M−C_7_H_7_O]^+^, C_4_H_8_N^+^).

When Kr^+^ was used as the reagent ion (Fig. 5(c)) only two significant product ions were observed in the mass spectra at *m*/*z* 72.08 which, as in case of NO^+^ and O_2_^+^, is the dominant fragment ion [M−C_7_H_5_O]^+^ and *m*/*z* 105.04 ([M−C_4_H_10_N]^+^, C_7_H_5_O^+^) which is observed at comparatively low relative abundances.

No significant contaminants were observed for reactions with any of the reagent ions, indicating that the supplied ethcathinone was reasonably pure.

There is little data available in the literature on the mass spectral analysis of ethcathinone, but the GC–MS analysis of dimethylcathinone, a close structural isomer of ethcathinone, is reported [26]. This shows a fragmentation pattern comparable to the observed results obtained when the analyte was ionised via charge transfer (NO^+^, O_2_^+^ and Kr^+^) with the most abundant ion observed at *m*/*z* 72. However, GC–MS also observed a significant peak at *m*/*z* 44 assigned to the loss of ethane from the fragment at *m*/*z* 72 which was not observed in the present study.

### 3.5 4-Methylethcathinone (4-MEC) and N-ethylbuphedrone (NEB)

The principle product ions observed when the structural isomers 4-MEC and NEB react with H_3_O^+^, NO^+^, O_2_^+^ and Kr^+^ have been discussed previously [8] but only at an *E*/*N* of 130 Td for H_3_O^+^, NO^+^ and O_2_^+^ and 95 Td for Kr^+^. Therefore we present here a much more extensive study with results covering a wide range in *E*/*N*.

#### 3.5.1 Reaction with H_3_O^+^

When H_3_O^+^ was used as the reagent ion at low *E*/*N* values (less than approximately 160 Td), the protonated molecular ion at *m*/*z* 192.14 ([MH]^+^, C_12_H_18_NO^+^) was the dominant product ion for both 4-MEC and NEB (Fig. 2(e) and (f)). Above 170 Td, *m*/*z* 174.14 ([MH−H_2_O]^+^, C_12_H_16_N^+^) became the dominant product ion. An ion with a constant branching ratio was observed at *m*/*z* 190.13 in both 4-MEC and NEB, this is assigned to [MH−H_2_]^+^ (C_12_H_16_NO^+^). The loss of H_2_ from the protonated parent molecule is a reaction channel that has been observed in previous studies dealing with illicit drugs with a similar structure [9]. At *E*/*N* > 135 Td, additional peaks were observed at *m*/*z* 146.10 and *m*/*z* 91.06 in both isomers; these masses are tentatively assigned to [MH−C_2_H_8_N]^+^ (C_10_H_10_O^+^) and [MH−C_5_H_11_NO]^+^ (C_7_H_7_^+^) respectively. In the investigation of NEB, an additional peak at *m*/*z* 118.07 was observed at *E*/*N* > 150 Td, which is assigned to [MH−C_4_H_12_N]^+^ (C_8_H_6_O^+^). A mass spectral peak observed at *m*/*z* 130.07 is assigned to result from a reaction with an impurity.

#### 3.5.2 Reactions with NO^+^, O_2_^+^ and Kr^+^

The most significant fragmentation pathway observed following the reaction of NEB with NO^+^ and O_2_^+^ is α cleavage at the ketone functional group leading to the formation of [M−C_7_H_5_O]^+^ at *m*/*z* 86.10 (C_5_H_12_N^+^) (Figs. 3(f) and 4(f)). This product ion could not be observed in the Kr^+^ system as it falls within the same *m*/*z* value as the very abundant ^86^Kr^+^ reagent ion. A second fragment ion was observed with ion branching ratios below 10%, at *m*/*z* 105.03 ([M−C_5_H_12_N]^+^, C_7_H_5_O^+^) when NEB was ionised by NO^+^ and O_2_^+^ via dissociative charge transfer and higher ion branching ratios for the reaction with Kr^+^ (Fig. 5(d)). Another product ion was observed at *m*/*z* 162.10 ([M−C_2_H_5_]^+^, C_10_H_12_NO^+^) for the reaction with Kr^+^. It should be noted that its branching ratio is artificially raised for the Kr^+^ data because we cannot determine the product ion signal strength at *m*/*z* 86.10. In the case of O_2_^+^ and NO^+^ the associated branching ratio for this product ion was less than 3% throughout the complete *E*/*N* range and hence is not included in that data.

The dominant product ion formed from the reaction of 4-MEC with NO^+^, O_2_^+^ (Figs. 3(e) and 4(e)) and the only observed product ion for Kr^+^ was *m*/*z* 72.08 ([M−C_8_H_7_O]^+^, C_4_H_10_N^+^), formed via α cleavage at the ketone functional group, the same mechanism that leads to the formation of the product ion [M−C_7_H_5_O]^+^ at *m*/*z* 86.10 in NEB. The product ion [M−C_4_H_10_N]^+^ at *m*/*z* 119.05, equivalent to *m*/*z* 105.03 in NEB, was observed at ion branching ratios below 10% in the NO^+^ system. In both the NO^+^ and O_2_^+^ systems the tropylium cation [M−C_5_H_10_NO]^+^ was observed at *m*/*z* 91.05 with its ion branching ratio increasing significantly with increasing *E*/*N*.

### 3.6 Ethylphenidate (EP)

#### 3.6.1 Reaction with H_3_O^+^

For H_3_O^+^ (Fig. 2(g)), the protonated parent molecule ([MH]^+^, C_15_H_22_NO_2_^+^, *m*/*z* 248.16) was the dominant product ion throughout the *E*/*N* range; its branching ratio began to decrease only at *E*/*N* > 200 Td, thus demonstrating a relatively high stability of the protonated species. Two principle fragment ions were detected, one at *m*/*z* 84.08 ([MH−C_10_H_12_O_2_]^+^, C_5_H_10_N^+^) and the other at *m*/*z* 165.09 ([MH−C_5_H_9_N]^+^, C_10_H_13_O_2_^+^); the latter was only observed for *E*/*N* < 135 Td. No significant organic impurities were detected. A similar fragmentation pattern was observed in previous studies, where Gas Chromatography–Electron Ionisation-Mass Spectrometry (GC–EI-MS) was used, although it is not possible to make a comparison of the branching ratios, due to the different nature of the ionisation methods [27].

#### 3.6.2 Reaction with NO^+^, O_2_^+^ and Kr^+^

No reaction with NO^+^ was observed, not even adduct formation. For O_2_^+^ and Kr^+^ dissociative charge transfer dominated and very high levels of fragmentation were observed, thus preventing identification of any characteristic peaks.

### 3.7 5-MeO-DALT

#### 3.7.1 Reaction with H_3_O^+^

Reaction with H_3_O^+^ (Fig. 2(h)) showed that the protonated parent molecule ([MH]^+^, C_17_H_23_N_2_O^+^, *m*/*z* 271.18) dominated for *E*/*N* < 185 Td; at higher *E*/*N* values *m*/*z* 174.09 ([MH−C_6_H_11_N]^+^, C_11_H_12_NO^+^, α-cleavage at the amino group) became the most abundant species. A third fragment ion, which was present at low branching ratios (<3%) at *E*/*N* < 155 Td but which increased to 10% by *E*/*N* 225 Td, was detected at *m*/*z* 160.08 ([MH−C_7_H_13_N]^+^, C_10_H_10_NO^+^). This fragment ion is most likely formed via β-cleavage at the amino group.

#### 3.7.2 Reaction with NO^+^, O_2_^+^ and Kr^+^

When NO^+^ was used as the reagent ion (Fig. 3(g)), at *E*/*N* values below 150 Td the dominant peak was observed at *m*/*z* 110.09 which is considered to be [M−C_10_H_10_NO]^+^ (C_7_H_12_N^+^), corresponding to a tertiary amine fragment. The branching ratio of the parent ion, *m*/*z* 270.19 ([M]^+^, C_17_H_22_N_2_O^+^), decreases from 25% to 6% as *E*/*N* is increased from 85 Td to 225 Td. Two additional ions were observed i.e. *m*/*z* 175.08 and *m*/*z* 159.06, tentatively identified as [M−C_6_H_9_N]^+^ (C_11_H_13_NO^+^) and [M−C_7_H_13_N]^+^ (C_10_H_9_NO^+^), respectively. When O_2_^+^ was used as the reagent ion (Fig. 4(g)), the parent ion peak was not observed, whilst *m*/*z* 175.08 and *m*/*z* 110.09 were, again, detected. Finally, when Kr^+^ was used as the reagent ion, dissociative charge transfer dominates. However, the very high levels of fragmentation observed prevent the identification of any characteristic peaks.

### 3.8 Dimethocaine

#### 3.8.1 Reaction with H_3_O^+^

Reaction of dimethocaine with H_3_O^+^ led to the production of 4 significant ions (Fig. 2(i)). Nondissociative proton transfer at *m*/*z* 279.20, ([MH]^+^, C_16_H_27_N_2_O_2_^+^), dominates across the whole *E*/*N* range investigated. At *E*/*N* greater than 185 Td fragment ions were observed at *m*/*z* 120.04 ([MH−C_9_H_21_NO]^+^, C_7_H_6_NO^+^) and 142.16 ([MH−C_7_H_7_NO_2_]^+^, C_9_H_20_N^+^) assigned to α and β cleavage at the ester functional group respectively. The product ion observed at *m*/*z* 86.10 remains at a constant low branching ratio for the whole *E*/*N* range investigated and is assigned to [MH−C_11_H_15_NO_2_]^+^ (C_5_H_12_N^+^).

Unidentified peaks observed in the mass spectra at *m*/*z* 307.24, *m*/*z* 265.19, *m*/*z* 251.18 and *m*/*z* 124.04, are attributed to reactions with unknown impurities in the sample. It has been suggested [3,28] that the synthesis of dimethocaine could proceed via the condensation of diethylamino-*t*-butanol and 4-aminobenzoic acid ethyl ester. The latter has a protonated parent molecule at *m*/*z* 166.09; this *m*/*z* was observed and therefore attributed to a reactant (or possibly an intermediate product) in the synthesis of dimethocaine.

Two additional ions at *m*/*z* 160.17 and *m*/*z* 138.06 are tentatively assigned to be C_9_H_22_NO^+^ and C_7_H_8_NO_2_^+^ respectively. These two ions may both be formed from proton transfer reactions with a neutral species formed by hydrolysis of dimethocaine producing 3-(diethylamino)-2,2-dimethylpropanol and 4-amino benzoic acid.

#### 3.8.2 Reactions with NO^+^, O_2_^+^ and Kr^+^

Following reaction with NO^+^ (Fig. 3(h)) the fragment ion [M−C_11_H_24_NO_2_]^+^ at *m*/*z* 86.10 becomes the dominant product ion across the *E*/*N* range investigated although the parent ion at *m*/*z* 278.19 ([M]^+^, C_16_H_26_N_2_O_2_^+^) is still detected at ion branching ratios below 10%. At *E*/*N* greater than 185 Td a mass spectral peak is seen at *m*/*z* 165.07 which is tentatively assigned to [M−C_7_H_15_N]^+^ (C_9_H_11_NO_2_^+^). The fragment ion at *m*/*z* 86.10 dominates throughout the *E*/*N* range investigated when O_2_^+^ is used as the reagent ion (Fig. 4(h)), with additional ions at *m*/*z* 149.03 and *m*/*z* 120.05 assigned to [M−C_8_H_19_N]^+^ (C_8_H_7_NO_2_^+^) and [M−C_9_H_20_NO]^+^ (C_7_H_6_NO^+^), respectively. Only one significant product ion is observed when Kr^+^ is used as the reagent ion at *m*/*z* 120.05, attributed to [M−C_9_H_20_NO]^+^ (C_7_H_6_NO^+^). No previous determination of the fragmentation pathways via chemical ionisation have come to our attention but a study of a related compound (procaine using positive-ion ESI) revealed similar fragmentation pathways [29] with α and β cleavage at the ester functional group also observed.

Following reactions with NO^+^, O_2_^+^ and Kr^+^, the most significant contaminant observed was at *m*/*z* 137.12 (C_7_H_7_NO_2_^+^) which agrees with the amino benzoic acid impurity found at *m*/*z* 138.06 from the H_3_O^+^ reaction. Additional ions resulting from reactions with impurities were observed at *m*/*z* 223.27 and 230.33 for NO^+^ and at *m*/*z* 53.01 in the O_2_^+^ system.

### 3.9 Nitracaine

Two batches with a different appearance, i.e. one in powder and one in fine crystal form, were analysed. The powder form of nitracaine did not show any mass spectral peak which could be attributed with any degree of certainty to nitracaine using any of the reagent ions, suggesting that it contained no active ingredient.

#### 3.9.1 Reaction of powder form with H_3_O^+^

In the case of H_3_O^+^, the principle mass spectral peak over the whole the *E*/*N* range was observed at *m*/*z* 160.17 (C_9_H_22_NO^+^). This is tentatively attributed to protonated 3-(diethylamino)-2,2-dimethylpropanol, a reagent which may be used in nitracaine synthesis [3,28]. Support for this assignment comes from the fact that this compound is a known irritant [30], and several online user forums mentioned that nasal irritation occurs following the use of nitracaine [2,3]. Additional ions were observed at *m*/*z* 86.10 (C_5_H_12_N^+^), *m*/*z* 142.10 (C_9_H_20_N^+^, loss of water from the amino alcohol) and *m*/*z* 158.15 (C_9_H_20_NO^+^, loss of H_2_ from the amino alcohol) and *m*/*z* 63.02, tentatively assigned to HNO_3_^+^.

#### 3.9.2 Reaction of powder form with NO^+^, O_2_^+^ and Kr^+^

When O_2_^+^ was used as a reagent ion, *m*/*z* 86.10 was again observed, this was the dominant ion. An additional ion was observed at *m*/*z* 128.14, tentatively attributed to C_8_H_18_N^+^, formed by the dissociative charge transfer reaction of O_2_^+^ with 3-(diethylamino)-2,2-dimethylpropanol, whilst two unidentified peaks, were detected at *m*/*z* 93.99 and 99.96. In the case of NO^+^, *m*/*z* 86.10 was again the dominant ion. Additional peaks with lower branching ratios were observed at *m*/*z* 142.10 (tentatively assigned to C_9_H_20_N^+^), *m*/*z* 158.15 (C_9_H_20_NO^+^) and again an unknown peak at *m*/*z* 93.99. Reaction with Kr^+^ shows no significant peaks in the mass spectra.

#### 3.9.3 Reaction of crystal form with H_3_O^+^

Many of the ions observed in the nitracaine powder form are also observed following reaction of the crystal form with H_3_O^+^ but in this case a mass spectral peak corresponding to the protonated molecule was observed at *m*/*z* 309.17 ([MH]^+^, C_16_H_25_N_2_O_4_^+^). This indicates that unlike the powder, the crystalline form contains nitracaine. Other ions were detected at *m*/*z* 160.17 (C_9_H_22_NO^+^, again the dominant mass spectral peak over the whole *E*/*N* range), *m*/*z* 86.10 (C_5_H_12_N^+^) and two unknown mass spectral peaks at *m*/*z* 107.05 and 101.02. Furthermore, a peak at *m*/*z* 63.02, corresponding to HNO_3_^+^, was observed.

#### 3.9.4 Reaction of crystal form with NO^+^, O_2_^+^ and Kr^+^

The only ions detected formed from the reaction of the reagent ion with impurities in the sample, as mentioned above for the powder form, but identical *m*/*z* values were not necessarily observed. Two ions, corresponding to C_5_H_12_N^+^ and C_7_H_4_NO_3_^+^, at *m*/*z* 86.10 and *m*/*z* 149.95, were detected for reactions with O_2_^+^ and NO^+^, with *m*/*z* 86.10 always being the most abundant. When O_2_^+^ was used as the reagent ion, an impurity at *m*/*z* 63.02, assigned to HNO_3_^+^ was detected; while after switching to NO^+^, an ion at *m*/*z* 142.10 (C_9_H_20_N^+^) became significant. When Kr^+^ was used as the reagent ion a large number of low intensity mass spectral peaks were observed preventing identification of any characteristic peaks.

The fact that the molecular parent ion was observed in the crystal but not in the powder form indicates that the supplied nitracaine in powder form contained nitracaine at insufficient levels to be detected (probably because of improper synthesis), thus explaining contradictory reports on the effect of nitracaine seen on online user forums [2,3].

## 4 Conclusions

Although some impurities were found in the drugs, all of the supplied compounds with the exception of nitracaine powder were found to contain the advertised active ingredient. This is surprising considering drugs counselling reports [31] on designer drugs (e.g. ecstasy = MDMA) purchased from the black market, which can contain virtually everything, from various mixtures of legal and illegal ingredients to common caffeine.

The fast pace of legislation designed to control new psychoactive substances leads to extremely short “invention to market” times and thus to increasing risks for the consumers; e.g. in the unfortunate case of the very recent UK ban on 5-APB, which was replaced by the presumably highly toxic 5-EAPB that has already caused fatalities among its first users [32]. However, the rapid spread of new psychoactive substances [33] means that the ability to rapidly detect and identify these species is gaining increasing importance for both policing and medical applications.

The reported product ion branching ratios formed from the analysis of a number of common new psychoactive substances (some, while legal at the time of purchase, are now controlled in some countries) by SRI-ToF-MS is designed to provide a reference for the development of techniques, capable of identifying these drug species in more complex ‘real world’ environments. These results add to the already large list of threat compounds detectable by SRI-ToF-MS. The potential role of this technology for the detection of threat agents has been established including research focusing on the detection of rape drugs in beverages [34]. This paves the way for the application of this technology to real-world situations, such as the detection of compounds adhered to surfaces (e.g. skin or fabrics) and the rapid identification of the constituents of blended drugs.

## Figures and Tables

**Table 1:** List of the investigated drugs (in order of increasing mass) and the product ions and their associated percentage ion branching ratios (in brackets) for reactions with H_3_O^+^, NO^+^, O_2_^+^ and Kr^+^, recorded at *E*/*N* of 130 Td (45 Td in case of Kr^+^). NR is used to represent no reaction and NOP means no observable product ions that can be identified to the drug as a result of substantial fragmentation.

Compound name	H_3_O^+^	NO^+^	O_2_^+^	Kr^+^
4-Fluoroamphetamine (C_9_H_12_FN; *m*/*z* 153.10)	*m*/*z* 154.10 – C_9_H_13_FN^+^ (19) *m*/*z* 137.07 – C_9_H_10_F^+^ (44) *m*/*z* 109.04 – C_7_H_6_F^+^ (37)	*m*/*z* 153.09 – C_9_H_12_FN^+^ (7) *m*/*z* 152.09 – C_9_H_11_FN^+^ (64) *m*/*z* 151.08 – C_9_H_10_FN^+^ (23) *m*/*z* 109.04 – C_7_H_6_F^+^ (6)	*m*/*z* 152.09 – C_9_H_11_FN^+^ (24) *m*/*z* 151.08 – C_9_H_10_FN^+^ (27) *m*/*z* 138.07 – C_8_H_9_FN^+^ (17) *m*/*z* 136.07 – C_9_H_9_F^+^ (11) *m*/*z* 109.04 – C_7_H_6_F^+^ (21)	*m*/*z* 152.09 – C_9_H_11_FN^+^ (26) *m*/*z* 138.07 – C_8_H_9_FN^+^ (34) *m*/*z* 109.04 – C_7_H_6_F^+^ (40)

Methiopropamine (C_8_H_13_NS; *m*/*z* 155.08)	*m*/*z* 156.08 – C_8_H_14_SN^+^ (36) *m*/*z* 125.04 – C_7_H_9_S^+^ (19) *m*/*z* 58.06 – C_3_H_8_N^+^ (45)	*m*/*z* 97.01 – C_5_H_5_S^+^ (1) *m*/*z* 58.06 – C_3_H_8_N^+^ (99)	*m*/*z* 97.01 – C_5_H_5_S^+^ (2) *m*/*z* 58.06 – C_3_H_8_N^+^ (98)	*m*/*z* 97.01 – C_5_H_5_S^+^ (6) *m*/*z* 73.05 – C_4_H_11_N^+^ (7) *m*/*z* 58.06 – C3H8N^+^ (87)

5-(2-Aminopropyl)benzofuran (C_11_H_13_NO; *m*/*z* 175.10)	*m*/*z* 176.11 – C_11_H_14_NO^+^ (14) *m*/*z* 159.08 – C_11_H_11_O^+^ (27) *m*/*z* 131.05 – C_9_H_7_O^+^ (59)	*m*/*z* 175.12 – C_11_H_13_NO^+^ (32) *m*/*z* 131.05 – C_9_H_7_O^+^ (68)	*m*/*z* 175.12 – C_11_H_13_NO^+^ (48) *m*/*z* 131.05 – C_9_H_7_O^+^ (52)	NOP

Ethcathinone (C_11_H_15_NO; *m*/*z* 177.12)	*m*/*z* 178.12 – C_11_H_16_NO^+^ (90) *m*/*z* 160.11 – C_11_H_14_N^+^ (10)	*m*/*z* 176.12 – C_11_H_14_NO^+^ (4) *m*/*z* 175.10 – C_11_H_13_NO^+^ (3) *m*/*z* 146.10 – C_10_H_12_N^+^ (3) *m*/*z* 105.04 – C_7_H_5_O^+^ (10) *m*/*z* 72.08 – C_4_H_10_N^+^ (80)	*m*/*z* 176.12 – C_11_H_14_NO^+^ (2) *m*/*z* 146.10 – C_10_H_12_N^+^ (2) *m*/*z* 107.05 – C_7_H_7_O^+^ (4) *m*/*z* 105.04 – C_7_H_5_O^+^ (9) *m*/*z* 77.04 – C_6_H_5_^+^ (2) *m*/*z* 72.08 – C_4_H_10_N^+^ (73) *m*/*z* 70.07 – C_4_H_8_N^+^ (8)	*m*/*z* 105.04 – C_7_H_5_O^+^ (4) *m*/*z* 72.08 – C_4_H_10_N^+^ (96)

4-Methylethcathinone (C_12_H_17_NO; *m*/*z* 191.13)	*m*/*z* 192.14 – C_12_H_18_NO^+^ (80) *m*/*z* 190.13 – C_12_H_16_NO^+^ (18) *m*/*z* 174.14 – C_12_H_16_N^+^ (2)	*m*/*z* 190.12 – C_12_H_16_NO^+^ (3) *m*/*z* 119.05 – C_8_H_7_O^+^ (1) *m*/*z* 72.08 – C_4_H_10_N^+^ (96)	*m*/*z* 119.05 – C_8_H_7_O^+^ (4) *m*/*z* 91.05 – C_7_H_7_^+^ (5) *m*/*z* 72.08 – C_4_H_10_N^+^ (91)	*m*/*z* 72.08 – C_4_H_10_N^+^ (100)

N-Ethylbuphedrone (C_12_H_17_NO; *m*/*z* 191.13)	*m*/*z* 192.14 – C_12_H_18_NO^+^ (84) *m*/*z* 190.13 – C_12_H_16_NO^+^ (13) *m*/*z* 174.14 – C_12_H_16_N^+^ (3)	*m*/*z* 190.12 – C_12_H_16_NO^+^ (3) *m*/*z* 105.03 – C_7_H_5_O^+^ (4) *m*/*z* 86.10 – C_5_H_12_N^+^ (93)	*m*/*z* 105.03 – C_7_H_5_O^+^ (9) *m*/*z* 86.10 – C_5_H_12_N^+^ (91)	*m*/*z* 162.10 – C_10_H_12_NO^+^ (40) *m*/*z* 105.03 – C_7_H_5_O^+^ (60)

Ethylphenidate (C_15_H_21_NO_2_; *m*/*z* 247.16)	*m*/*z* 248.16 – C_15_H_22_NO_2_^+^ (81) *m*/*z* 165.09 – C_10_H_13_O_2_^+^ (1) *m*/*z* 84.08 – C_5_H_10_N^+^ (18)	NR	NOP	NOP

5-MeO-DALT (C_17_H_22_N_2_O; *m*/*z* 270.13)	*m*/*z* 271.18 – C_17_H_23_N_2_O^+^ (91) *m*/*z* 174.09 – C_11_H_12_NO^+^ (6) *m*/*z* 160.08 – C_10_H_10_NO^+^ (3)	*m*/*z* 270.19 – C_17_H_22_N_2_O^+^ (14) *m*/*z* 175.08 – C_11_H_13_NO^+^ (15) *m*/*z* 159.06 – C_10_H_9_NO^+^ (26) *m*/*z* 110.09 – C_7_H_12_N^+^ (45)	*m*/*z* 175.08 – C_11_H_13_NO^+^ (52) *m*/*z* 110.09 – C_7_H_12_N^+^ (48)	NOP

Dimethocaine (C_16_H_26_N_2_O_2_; *m*/*z* 278.20)	*m*/*z* 279.20 – C_16_H_27_N_2_O_2_^+^ (91) *m*/*z* 142.16 – C_9_H_20_N^+^ (2) *m*/*z* 120.04 – C_7_H_6_NO^+^ (4) *m*/*z* 86.10 – C_5_H_12_N^+^ (3)	*m*/*z* 278.19 – C_16_H_26_N_2_O_2_^+^ (1) *m*/*z* 165.07 – C_9_H_11_NO_2_^+^ (3) *m*/*z* 86.10 – C_5_H_12_N^+^ (96)	*m*/*z* 149.03 – C_8_H_7_NO_2_^+^ (4) *m*/*z* 120.05 – C_7_H_6_NO^+^ (5) *m*/*z* 86.10 – C_5_H_12_N^+^ (91)	*m*/*z* 120.05 – C_7_H_6_NO^+^ (100)

Nitracaine (C_16_H_24_N_2_O_4_; *m*/*z* 308.17)	Crystal: *m*/*z* 309.17 – C_16_H_25_N_2_O_4_^+^ (100)	NR	NR	NOP
